# Clinical Features of Intraductal Papillary Mucinous Neoplasm-Related Pancreatic Carcinomas in Long-Term Surveillance

**DOI:** 10.3390/jcm14134585

**Published:** 2025-06-27

**Authors:** Kyohei Matsuura, Shinsaku Nagamatsu, Shoma Kikukawa, Yuya Nishio, Yusuke Komeda, Yuya Matsuo, Kohei Ohta, Chisa Yamamoto, Ayana Sueki, Kei Moriya

**Affiliations:** Department of Gastroenterology, Nara Prefecture General Medical Center, Nara 630-8581, Japan; graynurseshark07@kcn.jp (K.M.); joe.montana.no16@gmail.com (S.N.); ks50205020@gmail.com (S.K.); m12082yn@jichi.ac.jp (Y.N.); yusuke9827@gmail.com (Y.K.); jean.mat@outlook.jp (Y.M.); shacho3878@outlook.jp (K.O.); nmu114112cy@gmail.com (C.Y.); aya-sueki618@outlook.jp (A.S.)

**Keywords:** carcinogenesis, cohort studies, intraductal papillary mucinous neoplasm, pancreatic cyst, risk factors

## Abstract

**Background and Aims:** An appropriate surveillance system must be established to efficiently identify cases of intraductal papillary mucinous neoplasm (IPMN)-related malignant transformation. We analyzed the initial clinical background that affects long-term prognosis and narrowed the population for whom continued evaluation is inevitable. **Methods:** We included 1645 patients with IPMN treated at our hospital since 2010. We examined the types and timing of malignant transformation in terms of the worrisome features (WFs). The chi-squared test, log-rank test, and Cox proportional hazards model were used for the analysis (statistical significance at α = 0.05). **Results:** In total, 123 (7.5%) and 41 patients (2.5%) had IPMN-derived carcinoma (IPMN-DC) and concomitant pancreatic ductal adenocarcinoma (c-PDAC), respectively. Compared with IPMN-DC, a significantly higher proportion of c-PDAC patients were diagnosed with an advanced disease stage that developed earlier. The factors with significantly shorter time for IPMN-DC development were maximum cyst diameter (MCD) ≥ 30 mm, nonbranched type, main pancreatic duct (MPD) diameter ≥ 5 mm, and septal nodal structure (SNS) for IPMN-DC, and MCD ≥ 30 mm, main duct type, MPD ≥ 5 mm, SNS, cyst enlargement (≥2.5 mm/year), and abnormal CA19-9 levels for c-PDAC. Both groups could be significantly stratified by the number of WFs. A relative risk analysis revealed that SNS, MCD ≥ 30 mm, and MPD ≥ 5 mm were significant factors for IPMN-DC, whereas abnormal CA19-9 and SNS were significant for c-PDAC. Conversely, significantly more patients exhibiting these factors initially later developed IPMN-DC or c-PDAC. **Conclusions:** Ten percent of IPMN cases will develop IPMN-DC or c-PDAC, thereby requiring careful follow-up, especially in cases with SNS, abnormal CA19-9, and MCD ≥ 30 mm.

## 1. Introduction

With the development of imaging techniques, the frequency of the coincidental diagnosis of pancreatic cystic tumors has increased approximately 14-fold over the past 20 years [1]. In an analysis of 5296 physical examinations using magnetic resonance imaging (MRI), 13.7% of the examinees had pancreatic cystic tumors [2], and another observational study reported a detection rate of 2.1–10% by computed tomography (CT) [3,4]. The incidence of pancreatic cystic tumors increases with age and metabolic syndrome [2,3,5,6]. Considering that the incidence of pancreatic cystic tumors is positively correlated with patient age and that IPMNs occur most frequently among multifocal pancreatic cystic tumors [7], the number of IPMN patients shall increase in countries with aging populations.

IPMN is a cystic tumor originating in the pancreatic duct epithelium and is characterized by duct dilation and cyst formation due to mucus retention [7]. Although most IPMNs detected incidentally by various imaging tests have a tumor diameter of ≤10 mm and are considered to have a low risk of carcinogenesis [8], the possibility of malignant transformation is still a concern, and they are subject to periodic follow-up with imaging diagnosis. Therefore, an appropriate surveillance system should be set up to reliably and efficiently identify cases of IPMN-related malignant transformation, which are seen only in a limited number of patients with IPMN, within the framework of limited national healthcare funding [1]. In fact, the ideal interval between imaging tests, taking into account the physical and financial burden of IPMN patients, is an important clinical issue, and a large prospective randomized observational study has already begun, with results expected to be available in the near future [9]. The risk of pancreatic carcinogenesis in IPMNs is 5–10 times higher than that in normal subjects under long-term observation, even in the absence of malignant findings at diagnosis [10,11]. Furthermore, IPMN-related pancreatic tumors have two distinct pathologies: IPMN-derived carcinoma (IPMN-DC), which has a malignant component due to the primary lesion becoming larger over time, and concomitant pancreatic ductal adenocarcinoma (c-PDAC), which is not directly related to the primary lesion. Therefore, the entire pancreas should be evaluated, not just parts of the IPMN, during routine observation of IPMN [12]. However, c-PDAC can arise from small branched IPMNs without being related to the morphological features of the IPMN, and its early diagnosis is not always easy.

In this context, the concepts of worrisome features (WFs) and high-risk stigmata (HRS) have been proposed based on the accumulation of past scientific findings as a strategy to more efficiently identify cases that will develop malignant transformation [13]. These are a group of factors among the many clinical background factors in IPMN cases considered to be highly associated with cases of malignant transformation. In 2024, the international practice guidelines for IPMN were revised, and a modification from “cyst growth rate ≥5 mm per 2 years” to “cyst growth rate ≥2.5 mm per year” and new onset or acute exacerbation of diabetes within the past 1 year were added to WFs, and suspicious or positive results of cytology (if performed) were added to HRS [12]. This new guideline, the first revision in the last 7 years, adds to the body of evidence, including many of the above.

However, the question of which of these various clinical factors is most strongly associated with malignant transformation remains unanswered. Therefore, we designed and conducted this study to explore the clinical factors that predispose patients to malignant transformation, especially in IPMN patients.

## 2. Methods

### 2.1. Study Population and Surveillance of IPMNs

Among the patients who visited the Nara Prefecture General Medical Center between March 2010 and February 2023, those who had a history of (i) intraductal papillary mucinous adenocarcinoma of the pancreas (international classification of diseases 10th revision [ICD-10] code, C253; broad category, C00-D48; middle category, C15–C26), (ii) intraductal papillary mucinous adenoma of the pancreas (D136, C00-D48, D10–D36), (iii) intraductal papillary mucinous neoplasm of the pancreas (D377, C00-D48, D37–D48), (iv) serous cystadenocarcinoma of the pancreas (C259, C00–D48, C15–C26), (v) serous cystadenoma of the pancreas (D136, C00–D48, D10–D36), (vi) mucinous cystadenocarcinoma of the pancreas (C259, C00–D48, C15–C26), (vii) mucinous cystadenoma of the pancreas (D136, C00–D48, D10–D36), (viii) neoplastic pancreatic cyst (D377, C00–D48, D37–D48), (ix) serous cystic tumor of the pancreas (D377, C00–D48, D37–D48), (x) mucinous cystic tumor of the pancreas (D377, C00–D48, D37–D48], and (xi) pancreatic cyst (K862, K00–K93, K80–K87), were automatically selected by the electronic medical record system (n = 2152). On the other hand, patients without IPMN and without detailed medical information were excluded in this study. All the patients were followed until 29 February 2024, or death, whichever came earlier. Patients with IPMN were classified into the following three different morphological phenotypes. First, the “branch-duct IPMNs” are defined as unilocular or multilocular pancreatic cystic lesions that communicate with the main pancreatic duct (MPD). Second, the “main-duct IPMNs” are defined as segmental or diffuse dilatation of the MPD of >5 mm without other causes of MPD dilatation. Third, the “mixed-type IPMNs” are defined as lesions meeting the diagnostic criteria for both branch-duct and main-duct IPMNs [7].

All the patients underwent routine laboratory examinations, including general biochemistry test, carcinoembryonic antigen (CEA), and carbohydrate antigen 19-9 (CA19-9), every 6–12 months based on the previous IPMN international consensus guidelines [13,14,15]. The normal upper limits of the serum CA19-9 and CEA concentrations in this study were 37 U/mL and 5.0 ng/mL, respectively. Imaging tests were also performed, including MRI and/or contrast-enhanced CT. In case of any signs raising suspicion of pancreatic carcinoma development on these imaging modalities, endoscopic ultrasound-guided fine-needle aspiration (EUS-FNA) and/or endoscopic retrograde cholangiopancreatography (ERCP) were performed to confirm cytological or histological diagnosis of pancreatic carcinoma [16]. EUS-FNA was not performed in principle for the cytological analysis of the cyst fluid based on the local consensus in Japan [12]. Not only invasive carcinoma but also IPMN with high-grade dysplasia were regarded as malignant tumors and analyzed as IPMN-related carcinoma. In patients with IPMN-related carcinoma, those with IPMN-DC and concomitant PDAC were differentiated based on the radiological and/or pathological results.

### 2.2. Evaluation of the Morphological Features of IPMNs in the Prediagnostic Stage of Pancreatic Carcinoma

Based on the current international consensus guideline [12], we characterized the morphologic features of IPMNs before achieving a pancreatic carcinoma diagnosis focusing on WFs and HRS. To be specific, the former includes (i) an IPMN size ≥ 30 mm, (ii) an enhancing mural nodule < 5 mm, (iii) a thickened enhanced cyst wall, (iv) an MPD diameter of 5–9.9 mm, (v) an abrupt caliber change of the MPD with distal pancreatic atrophy, (vi) lymphadenopathy, (vii) a cyst growth rate ≥ 2.5 mm/year, (viii) acute pancreatitis, (ix) increased serum CA19-9 level (normal upper limit, 37 U/mL), and (x) new onset or acute exacerbation of diabetes within the past 1 year, and the latter includes (i) obstructive jaundice in a patient with IPMN at pancreatic head, (ii) an enhancing mural nodule ≥ 5 mm or solid component, (iii) MPD diameter ≥ 10 mm, and (iv) suspicious or positive results of cytology (if performed). A further examination via endoscopic ultrasound is recommended for IPMNs harboring WFs and surgical resection for IPMNs harboring HRS.

The distinction between IPMN-DC and c-PDAC is based on the continuity of the localization of the solid component that is suspected to be an invasive carcinoma and the mural nodule in the cyst of the IPMN on imaging and histological transition between the IPMN and invasive pancreatic ductal carcinoma on pathological examination.

### 2.3. Definition of Serious Medical Event and Active Medical Intervention

We defined “serious medical event” as a condition wherein surgical resection of the target tumor is absolutely recommended based on histological diagnostic evidence and diagnostic imaging confirmation, or a condition wherein surgical resection is necessary but difficult due to distant metastasis or poor general condition. Moreover, we defined “active medical intervention (AMI)” as surgical intervention for serious medical events or follow-up with the best supportive care after thorough consultation. The rate and timing of AMI were set as the main items for analysis in this study.

### 2.4. Statistical Analyses

Pearson’s chi-squared test was used to assess unrelated categorical variables. The log-rank test was adopted to determine the significant differences between the Kaplan–Meier curves. Numerical variables are shown as the median with interquartile ranges. Two-sided *p* < 0.05 indicated statistically significant differences. The EZR software (version 1.68) program, a graphical user interface for the R (version 4.3.1) statistical computing and graphics environment [17], was used for statistical analyses. JMP version 14.3 (SAS Institute Inc., Cary, NC, USA) software was also used.

### 2.5. Ethical Issues

This study was conducted following the Declaration of Helsinki and the principles of the Japanese ethics guideline for life science and medical research involving human subjects (https://www.mext.go.jp/lifescience/bioethics/files/pdf/n2373_01.pdf [Only Japanese text available], accessed on 28 March 2025). Furthermore, the Ethical Committee of Nara Prefecture General Medical Center approved this study (approval number: 983). Informed consent was obtained by using an opt-out method.

## 3. Results

### 3.1. Clinical Characteristics of the Entire Study Population at the Initial Diagnosis of IPMN

After excluding patients without IPMN (n = 499) and those without detailed medical information (n = 8), 1645 patients with IPMN were finally enrolled in the current retrospective cohort study (Supplementary Figure S1). In this study, we conducted a detailed investigation of the clinical course of 1645 patients with IPMN who had attended our institution for up to 20 years (median observation period was 36 months) and obtained the following novel findings by analyzing a huge amount of data totaling 7000 person-years. The largest IPMN lesions were found in the pancreatic body (36.6%), pancreatic head (36.1%), and pancreatic tail (19.2%), and the median diameter of the largest cyst was 13.0 mm at initial examination (Table 1). The most common IPMN type was the branched type (93.2%), and the main pancreatic duct and mixed types accounted for 4.2% and 2.6%, respectively. The median maximum MPD diameter was 2.0 mm, and findings of enhancing mural nodules and cyst walls, which constitute HRS, were identified in 183 patients (11.1%).

### 3.2. Clinical Characteristics of the Populations with IPMN-DC, c-PDAC, and Routine Follow-Up at the Initial Diagnosis of IPMN

**Figure 1:**
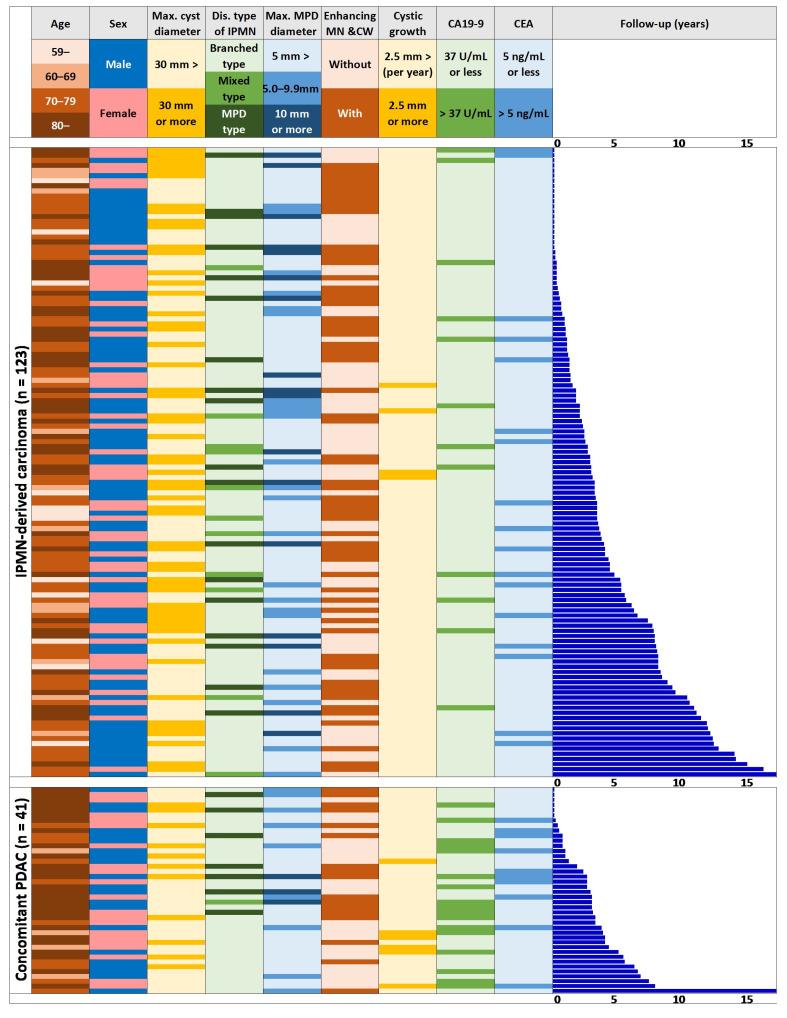
Clinical features at initial presentation of the cases with IPMN-DC and concomitant PDAC. PDAC, pancreatic ductal adenocarcinoma.

Subsequently, we analyzed all eligible patients with IPMN, dividing them into those followed up without the need for medical intervention (n = 1481), those who later developed IPMN-DC (n = 123), and those who had c-PDAC (n = 41). In the IPMN-DC group, the tumor incidence was highest among those in their 70s but was evenly distributed among all the age groups, whereas in the c-PDAC group, an increase with age was noted (Table 2, Figure 1). Additionally, the IPMN-DC group had significantly more males, but no gender differences were found in the c-PDAC group. Both of these patient groups had a larger maximum cyst diameter, the highest frequency of MPD type, a larger MPD diameter, and significantly higher percentages of enhancing mural nodules and cyst walls during initial IPMN diagnosis. Conversely, no significant difference was found in the definite cyst diameter increase (≥2.5 mm/year) or tumor marker abnormalities in the IPMN-DC group, whereas the c-PDAC group had a significantly higher proportion of definite cyst diameter increase (≥2.5 mm/year) and a higher proportion of tumor marker abnormalities (Table 2, Figure 1).

### 3.3. Cumulative Incidence Rate of IPMN-DC Classified by Clinical Factors

**Figure 2:**
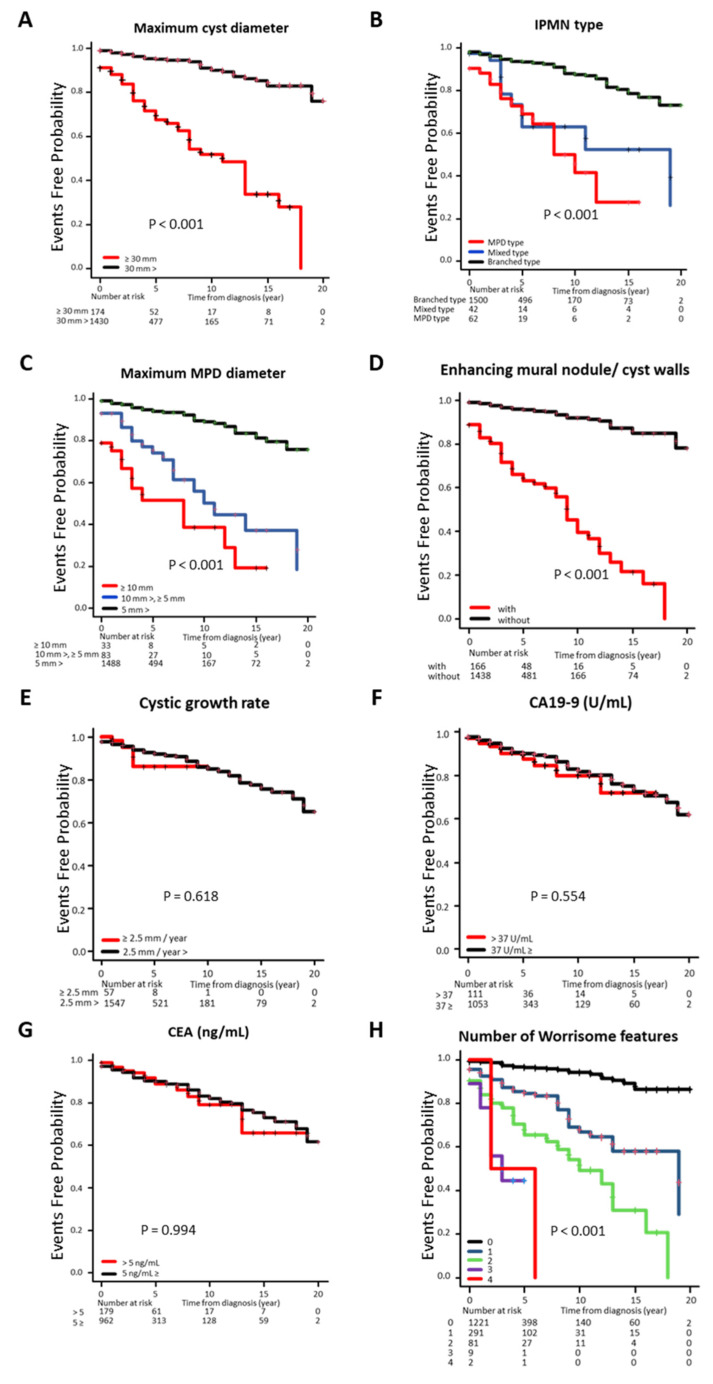
Cumulative incidence rate of concomitant IPMN-DC classified by clinical factors. (**A**) maximum cyst diameter, (**B**) IPMN type, (**C**) maximum MPD diameter, (**D**) enhancing mural nodule and cyst walls, (**E**) cystic growth rate, (**F**) CA19-9, (**G**) CEA, and (**H**) number of WFs. CA19-9, carbohydrate antigen 19-9; CEA, carcinoembryonic antigen; IPMN, intraductal papillary mucinous neoplasms; WF, worrisome feature.

Of the 164 patients (10.0%) requiring medical intervention during the study, 123 (7.5%) had IPMN-DC, and 1604 (123 and 1481 patients who were followed up) were included in the Kaplan–Meier survival curve analysis (Figure 2). The time from the initial diagnosis to the time of medical intervention was significantly shorter (*p* < 0.001) in cases with a maximum tumor diameter of ≥30 mm compared with those with a maximum tumor diameter of <30 mm (Figure 2A). Additionally, the branched-type IPMN was significantly longer than the other types (*p* < 0.001). In the classification by MPD diameter, an inverse correlation was found between duration and diameter size (*p* < 0.001) (Figure 2B,C). The duration was shorter in the group with enhancing mural nodules and cyst walls (*p* < 0.001) (Figure 2D), but the rate of cyst diameter increase or abnormal tumor marker level was not significantly different (Figure 2E–G). The analysis by the number of WFs showed a significant inverse correlation with the number of corresponding WFs (*p* < 0.001) (Figure 2H).

### 3.4. Cumulative Incidence Rate of c-PDAC Classified by Clinical Factors

**Figure 3:**
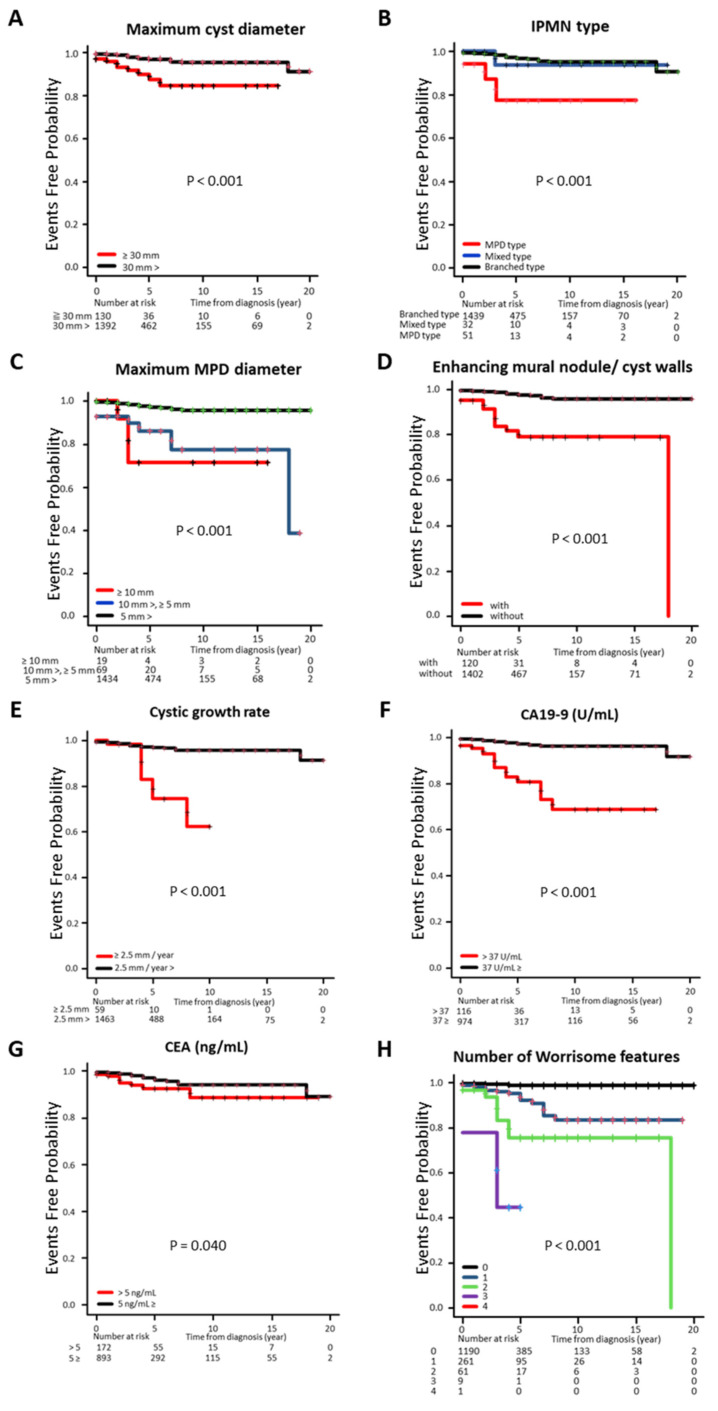
Cumulative incidence rate of concomitant PDAC classified with clinical factors. (**A**) maximum cyst diameter, (**B**) IPMN type, (**C**) maximum MPD diameter, (**D**) enhancing mural nodule and cyst walls, (**E**) cystic growth rate, (**F**) CA19-9, (**G**) CEA, and (**H**) number of WFs. CA19-9, carbohydrate antigen 19-9; CEA, carcinoembryonic antigen; PDAC, pancreatic ductal adenocarcinoma; WF, worrisome feature.

Of the 164 patients (10.0%) requiring medical intervention during the study, 41 (2.5%) had c-PDAC, and 1522 patients (41 and 1481 patients who were followed up) were included in the Kaplan–Meier survival curve analysis (Figure 3). The time from initial diagnosis to medical intervention was significantly shorter (*p* < 0.001) in cases with a maximum tumor diameter of ≥30 mm compared with those with a maximum tumor diameter of <30 mm (Figure 3A). The MPD type was significantly shorter than the other IPMN types (*p* < 0.001). In the classification by MPD diameter, an inverse correlation was found between duration and diameter size (*p* < 0.001) (Figure 3B,C). The duration was shorter in the group with enhancing mural nodules and cyst walls (*p* < 0.001), with a larger rate of cyst diameter increase and with abnormal CA19-9 levels (Figure 3D–F), but the abnormal CEA levels were not significantly different (Figure 3G). The analysis by the number of WFs showed a significant inverse correlation with the number of corresponding WFs (*p* < 0.001) (Figure 3H).

### 3.5. Differences in the Timing of Diagnosis and Clinical Staging of Pancreatic Neoplasms Associated with IPMN

The time required to develop c-PDAC was significantly shorter than that for IPMN-DC (*p* = 0.049), and >80% of the former cases developed within 5 years, whereas in the latter, there were scattered cases that developed after the 10th year (Supplementary Figure S2). In terms of staging during the definitive diagnosis of pancreatic neoplasms, 118 of 123 patients (95.9%) in the IPMN-DC group were diagnosed at an early stage of Stage II or below, whereas 12 of 41 patients (29.2%) in the c-PDAC group were diagnosed at an advanced stage (*p* < 0. 001) (Supplementary Table S1).

### 3.6. Risk Factor Analysis for the Development of IPMN-DC and c-PDAC

Subsequently, we assessed the impact of risk factors on the development of pancreatic neoplasms. Before conducting the Cox proportional hazards model analysis for this purpose, we individually assessed whether the proportional hazard was maintained for each risk factor, mainly based on the Kaplan–Meier survival curves in Figure 2 and Figure 3. In the IPMN-DC group, these risk factors were sex, maximum cyst diameter (≥30 mm), IPMN type (MPD including mixed type), maximum MPD diameter (≥5 mm), and the presence of enhancing mural nodules or cyst walls. Similarly, in the c-PDAC group, we used the following criteria: age, maximum cyst diameter (>30 mm), IPMN type (MPD type), maximum MPD diameter (>5 mm), the presence of enhancing mural nodules or cyst walls, and serum CA19-9 concentration.

Therefore, a Cox proportional hazards model analysis was performed for these factors, and the largest hazard factor for IPMN-DC was the presence of enhancing mural nodules or cyst walls (hazard ratio, 5.2; 95% confidence interval, 3.5–7.7) (Table 3). A maximum cyst diameter >30 mm (hazard ratio, 3.3; 95% CI, 2.3–4.9) and a maximum MPD diameter > 5 mm (hazard ratio, 2.6; 95% CI, 1.6–4.3) were also extracted as significant hazard factors. By contrast, for c-PDAC, an abnormally high CA19-9 level was the largest hazard factor (hazard ratio, 5.0; 95% CI, 2.5–10.0), and having enhancing mural nodules or cyst walls was the other significant hazard factor (hazard ratio, 4.8, 95% CI, 2.3–10.0) (Table 3).

### 3.7. Frequency of Pancreatic Malignant Neoplasms by Each WF at the Initial Diagnosis of IPMN

We performed a reverse analysis at the end to increase confidence in the above results. Supplementary Figure S3A shows that a significantly higher percentage of patients exhibiting enhancing mural nodule or cyst walls, maximum cyst diameter ≥ 30 mm, and main pancreatic duct diameter ≥ 5 mm at initial presentation, developed IPMN-DC later. Additionally, a significantly larger population of patients exhibiting enhancing mural nodule or cyst walls, maximum cyst diameter ≥30 mm, main pancreatic duct diameter ≥5 mm, and abnormally high CA19-9 levels at initial presentation, also developed c-PDAC later (Supplementary Figure S3B).

## 4. Discussion

Two different types of pancreatic malignant neoplasms occur in patients with IPMN: IPMN-DC, wherein IPMN invades the pancreatic parenchyma, and c-PDAC, wherein noncontiguous invasive pancreatic ductal carcinoma develops in the pancreas, where the IPMN originated. The cumulative incidence of IPMN-DC and c-PDAC actually differs between reports [11,18,19]. One reason for the differences may be the hospital location affecting referral rates from other hospitals. Another reason may be some difficult cases in distinguishing between the two, and the existence of cases classified as IPMN-DC cannot be denied based on the understanding that they are malignant pancreatic neoplasms that have developed in patients with IPMN. In any case, we will wait for the accumulation of reports to finally conclude on the ratio for the histological classification of malignant neoplasms occurring in IPMN cases.

The importance of efficiently identifying high-risk cases among patients with IPMN has recently been widely recognized, and the components of WF and HRS, which are strongly associated with the development of pancreatic malignant neoplasms, were revised in the IPMN international practice guidelines, which were updated after 7 years [12]. Specifically, the new HRS category is “positive or suspicious cytology” and the new WSF category is “new-onset diabetes mellitus or acute exacerbation of existing diabetes mellitus.” Unfortunately, due to the low rate of EUS-FNA in this study, we were unable to study the cytology results included in the HRS. We purposely excluded four WS items from the present analysis: recurrent pancreatitis, new-onset diabetes mellitus, ductal caliber discrepancy, and lymph node enlargement. This is because the definitions of these are unclear and difficult to objectively classify. It is recognized that the onset of diabetes is difficult to define clearly, and there is no certain consensus on the duration and severity of recurrent pancreatitis [20,21]. Setting cutoff values is also difficult for ductal caliber discrepancy and lymph node enlargement. Evidently, these four items are important clues to suspect new onset of pancreatic cancer in daily practice. Approximately 20% of IPMN cases have been reported to have a history of recurrent acute pancreatitis [12,22,23]. Therefore, it would contribute to a more accurate prognosis if pancreatitis and diabetes mellitus were to be clearly defined and included in the evaluation items, and the same is true for the treatment of pancreatic duct diameter discrepancies and lymph node enlargement.

Although we performed the analysis under limited conditions, we showed a variety of findings. Specifically, among pancreatic malignant neoplasms, IPMN-DC tends to be predominantly male, whereas patients with c-PDAC tend to show abnormally high CA19-9 and CEA levels already on their initial presentation of IPMN. Moreover, this study demonstrated that risk factors for IPMN-DC and c-PDAC are a maximum cyst diameter of 30 mm, nonbranched type, main pancreatic duct dilatation of 5 mm, and a cystic-walled nodule. Wood et al., identified the risk factors for the development of c-PDAC, including women and nondilatation of the main pancreatic duct, in addition to hyperechogenic pancreas and advanced age [24]. Oyama et al. found that the diameter of the dilated pancreatic duct and the nodule size in the wall were irrelevant for the development of c-PDAC [10]. Although these reports conflict with those of the present analysis, the results may have been affected by the former being a review article, which thus enrolled different groups of patients with different backgrounds, and the latter being limited to branched IPMN.

Furthermore, we found a positive correlation between the number of WFs and the frequency of occurrence of medical intervention, and cases with at least one WF were associated with the need for medical intervention during the long-term course of the disease. In 49 (29.9%) of 164 cases requiring medical intervention, medical intervention was performed after ≥5 years, and, therefore, the revised guideline proposed that “cases with a cyst diameter of <20 mm and no significant change in 5 years can be considered for termination of follow-up observation” [12] should be considered by the physician in charge. Additionally, the current guideline emphasizes risk management for IPMN-DC, and additional consideration from the perspective of strengthening risk management for c-PDAC is desirable. Furthermore, the very important issue of over-treatment or over-diagnosis should be discussed. In this study, tumor resection was performed in 118 of 123 patients with an imaging diagnosis of IPMN-DC, but only 35 (29.7%) of these patients had a final pathologic diagnosis of adenocarcinoma or high-grade dysplasia. In other words, from a retrospective point of view, about 70% of cases of suspected IPMN-DC were consequently over-treated or over-diagnosed. The existence of this discrepancy is the difficulty in correctly diagnosing IPMN-related tumors (especially IPMN-DC), and this fact needs to be explained to patients with IPMN.

This study has several limitations that should be considered. First, the limited number of patients included in this study and the presence of selection bias are important limitations. However, we analyzed >1600 patients, and the fact that this was a single-center study made it possible to collect accurate and uniform information. Second, the imaging evaluation with EUS was not available for all patients, and EUS-FNA was even less commonly performed, because the results are highly dependent on the level of cytology and molecular marker analysis at the facility and the skill of the endoscopist. Furthermore, EUS-FNA for pancreatic cystic lesions is not performed in Japan in principle due to the risk of seeding. But, in the future, we would like to investigate the characteristics of the mural nodules and the solid component in detail by EUS to determine the position of HRS in IPMN cases, because the sensitivity of EUS for detecting intracystic mural nodules, which are most significantly involved in the development of IPMN-DC and c-PDAC, is >90%, surpassing the sensitivity of CT and MRI [25,26]. Third, we analyzed the entire course of HRS and WF with evaluation findings limited to the initial examination. In other words, the cumulative effect of HRS and WF on new cases of HRS and WF during the disease course after the initial diagnosis was not evaluated, and this point should also be clarified. Fourth, the endpoint of this study was set at the presence or absence of AMI for the occurrence of a serious medical event. Thus, some cases of surgical tumor resection, wherein an obvious malignant neoplasm could not be proven pathologically, and some cases wherein pathological diagnostic approaches were omitted because of old age or reduced activity of daily life, although a pancreatic malignant neoplasm was very strongly suspected based on multiple clinical findings. We should also consider that the study results were not examined with respect to overall survival. Fifth, we did not analyze jaundice in the HRS. Only one case (0.06%) had an abnormal direct bilirubin level and a total bilirubin level of ≥2.0 mg/dL, and he had been diagnosed with IPMN-DC early after his visit and had undergone surgical resection. Sixth, insufficient information on the presence or absence of a family history of pancreatic malignant neoplasms made it difficult to reveal its influence. Seventh, an analysis of hemoglobin A1c as well as pancreatic enzymes, which might influence the malignant transformation of IPMN, was desired, but unfortunately, the very large number of missing measurements made it impossible to generate sufficiently reliable data.

However, despite these limitations, this study successfully supported the usefulness of the current international guideline for IPMN and revealed the remaining issues to be resolved. Thus, network meta-analyses and multicenter prospective observational studies should be conducted to further improve the accuracy of the facts obtained to date. Consequently, the stratification of pancreatic carcinogenesis risk based on a detailed understanding of the clinical backgrounds of eligible patients using a questionnaire developed based on uniform and specific criteria for clinical signs will contribute significantly to reducing the burden on healthcare financing in each country.

In conclusion, this study successfully revealed that the presence of enhancing mural nodule/cyst walls could be a significant risk factor for malignant transformation of IPMN. In addition, maximum tumor diameter over 30 mm, for IPMN-DC, and abnormally high level of CA19-9, for PDAC, could be serious risks.

## Figures and Tables

**Table 1:** Clinical profiles of enrolled cases on initial diagnosis of IPMN (n = 1645).

Age (Years Old)	79.0 [71.0–85.0]
**Sex**	
Male	776 (47.2%)
Female	869 (52.8%)
**Follow-up periods (year)**	3.0 [0.0–6.0]
**Disease location**	
Uncus	132 (8.1%)
Head	595 (36.1%)
Body	602 (36.6%)
Tail	316 (19.2%)
**Maximum cyst diameter (mm)**	13.0 [7.0–20.0]
**Disease type of IPMN**	
MPD type	69 (4.2%)
Branched type	1533 (93.2%)
Mixed type	43 (2.6%)
**Maximum MPD diameter (mm)**	2.0 [1.6–3.0]
**Enhancing mural nodule/cyst walls**	
With	183 (11.1%)
Without	1462 (88.9%)
**Cystic growth rate (mm/year)**	
≥2.5	63 (3.8%)
2.5>	1582 (96.2%)
**CA19-9 (U/mL)**	10.9 [6.5–20.2]
**CEA (ng/mL)**	2.5 [1.6–4.0]

CA19-9, carbohydrate antigen 19-9; CEA, carcinoembryonic antigen; IPMN, intraductal papillary mucinous neoplasms; IQR, interquartile range; MPD, main pancreatic duct. Continuous variables are presented as median and IQR, while categorical variables are presented as number of cases and percentage.

**Table 2:** Clinical profiles of patients with routine follow-up, IPMN-DC, and concomitant PDAC on initial diagnosis of IPMN.

	Routine Follow-Up (n = 1481)	IPMN-DC (n = 123)		Concomitant PDAC (n = 41)	
Clinical Factors	(Percent, Cases)	(Percent, Cases)	*p*	(Percent, Cases)	*p*
**Age**					
–59	9.1% (135/1481)	9.8% (12/123)	<0.001	0.0% (0/41)	0.081
60–69	13.4% (199/1481)	9.8% (12/123)		9.8% (4/41)	
70–79	28.6% (424/1481)	47.2% (58/123)		24.4% (10/41)	
80–	48.8% (723/1481)	33.3% (41/123)		65.9% (27/41)	
**Sex**					
Male	46.0% (682/1481)	59.3% (73/123)	<0.01	51.2% (21/41)	0.51
Female	54.0% (799/1481)	40.7% (50/123)		48.8% (20/41)	
**Maximum cyst diameter**					
≥30 mm	8.0% (119/1481)	44.7% (55/123)	<0.0001	26.8% (11/41)	<0.01
30 mm>	92.0% (1362/1481)	55.3% (68/123)		73.2% (30/41)	
**Disease type of IPMN**					
MPD type	3.0% (44/1481)	14.6% (18/123)	<0.0001	17.1% (7/41)	<0.0001
Mixed type	2.1% (31/1481)	8.9% (11/123)		2.4% (1/41)	
Branched type	94.9% (1406/1481)	76.4% (94/123)		80.5% (33/41)	
**Maximum MPD diameter**					
≥10 mm	1.1% (16/1481)	13.8% (17/123)	<0.0001	7.3% (3/41)	<0.0001
10 mm>, ≥5 mm	4.1% (60/1481)	18.7% (23/123)		22.0% (9/41)	
5 mm>	94.9% (1405/1481)	67.5% (83/123)		70.7% (29/41)	
**Enhancing MN/cyst walls**					
With	7.0% (103/1481)	51.2% (63/123)	<0.0001	41.5% (17/41)	<0.0001
Without	93.0% (1378/1481)	48.8% (60/123)		58.5% (24/41)	
**Cystic growth rate**					
≥2.5 mm/year	3.6% (53/1481)	3.3% (4/123)	1.00	14.6% (6/41)	<0.001
2.5 mm/year>	96.4% (1428/1481)	96.7% (119/123)		85.4% (35/41)	
**CA19-9**					
>37 U/mL	9.4% (99/1053)	10.8% (12/111)	0.63	45.9% (17/37)	<0.0001
37 U/mL≥	90.6% (954/1053)	89.2% (99/111)		54.1% (20/37)	
**CEA**					
>5 ng/mL	15.7% (162/1030)	15.3% (17/111)	0.91	28.6% (10/35)	<0.05
5 ng/mL≥	84.3% (868/1030)	84.7% (94/111)		71.4% (25/35)	

CA19-9, carbohydrate antigen 19-9; CEA, carcinoembryonic antigen; IPMN-DC, intraductal papillary mucinous neoplasms derived carcinoma; IQR, interquartile range; MN, mural nodule; MPD, main pancreatic duct; PDAC, pancreatic ductal adenocarcinoma. Continuous variables are presented as median and IQR, while categorical variables are presented as number of cases and percentage.

**Table 3:** Clinical factors affecting the development of pancreatic neoplasms.

IPMN-DC	Hazard Ratio [95% CI]	***p***
Enhancing mural nodule/cyst walls (with)	5.18 [3.47–7.74]	<0.0001
Maximum cyst diameter (≥30 mm)	3.33 [2.25–4.92]	<0.0001
Maximum MPD diameter (≥5 mm)	2.61 [1.62–4.25]	<0.0001
Sex (male)	1.14 [0.78–1.65]	0.38
Disease type of IPMN (nonbranched type)	1.17 [0.69–1.97]	0.52
**Concomitant PDAC**	Hazard Ratio [95% CI]	*p*
CA19-9 (>37 U/mL)	5.02 [2.51–10.0]	<0.0001
Enhancing mural nodule/cyst walls (with)	4.76 [2.25–10.0]	<0.0001
Maximum MPD diameter (≥5 mm)	2.23 [0.90–5.54]	0.08
Maximum cyst diameter (≥30 mm)	1.94 [0.90–4.17]	0.09
Age (years old)	1.02 [0.98–1.06]	0.40
Disease type of IPMN (nonbranched type)	1.25 [0.45–3.43]	0.67

CA19-9, carbohydrate antigen 19-9; CI, confidence interval; IPMN-DC, intraductal papillary mucinous neoplasms derived carcinoma; MPD, main pancreatic duct, PDAC, pancreatic ductal adenocarcinoma.

## Data Availability

All the data used to support the findings of this study are included in the article.

## Supplementary Materials

The following supporting information can be downloaded at: https://www.mdpi.com/article/10.3390/jcm14134585/s1, Figure S1: Patient flow chart in the current study. Figure S2: Post-diagnosis cumulative incidence of IPMN-DC or concomitant PDAC. Figure S3: Frequency of pancreatic malignant neoplasms by each worrisome feature at the initial diagnosis of IPMN. Table S1: Clinical tumor stage of pancreatic neoplasms in patients with IPMN.

## Abbreviations

AMI	Active medical intervention
CT	Computed tomography
HRS	High-risk stigmata
IPMN	Intraductal papillary mucinous neoplasm
MPD	Main pancreatic duct
MRI	Magnetic resonance imaging
SNS	Septal nodal structure
WF	Worrisome feature
