# Supplementary material for: Clinical Features of Intraductal Papillary Mucinous Neoplasm-Related Pancreatic Carcinomas in Long-Term Surveillance
Source: J Clin Med. 2025 Jun 27;14(13):4585. doi: 10.3390/jcm14134585 (PMC12249908; doi:10.3390/jcm14134585)
Supplement: Supplementary file 1 [file jcm-14-04585-s001.zip › Supplementary figure legends.pdf]

Supplementary Figure legends

Supplementary Figure S1 Patient flow chart in the current study. We initially enrolled 1645 patients with IPMN, and 164 of them who developed either IPMN-DC or concomitant PDAC were carefully examined in detail. IPMN, intraductal papillary mucinous neoplasms; PDAC, pancreatic ductal adenocarcinoma.

Supplementary Figure S2 Post-diagnosis cumulative incidence of IPMN-DC or concomitant PDAC. IPMN, intraductal papillary mucinous neoplasms; PDAC, pancreatic ductal adenocarcinoma.

Supplementary Figure S3 Frequency of pancreatic malignant neoplasms by each worrisome feature at the initial diagnosis of IPMN. (A) IPMN-DC, (B) concomitant PDAC. CA19-9, carbohydrate antigen 19-9; CW, cyst wall; IPMN, intraductal papillary mucinous neoplasms; MN, mural nodule; MPD, main pancreatic duct; PDAC, pancreatic ductal adenocarcinoma.
