# Supplementary material for: Clinical Features of Intraductal Papillary Mucinous Neoplasm-Related Pancreatic Carcinomas in Long-Term Surveillance
Source: J Clin Med. 2025 Jun 27;14(13):4585. doi: 10.3390/jcm14134585 (PMC12249908; doi:10.3390/jcm14134585)
Supplement: Supplementary file 1 [file jcm-14-04585-s001.zip › Supplementary Methods.pdf]

Supplementary Methods

Study population and classification of IPMNs

Among the patients who visited our institution between March 2010 and February 2023, those who had a history of the following disease: (i) intraductal papillary mucinous adenocarcinoma of the pancreas (international classification of diseases 10th revision [ICD-10] code, C253; broad category, C00-D48; middle category, C15–C26), (ii) intraductal papillary mucinous adenoma of the pancreas (D136, C00-D48, D10–D36), (iii) intraductal papillary mucinous neoplasm of the pancreas (D377, C00-D48, D37–D48), (iv) serous cystadenocarcinoma of the pancreas (C259, C00-D48, C15–C26), (v) serous cystadenoma of the pancreas (D136, C00–D48, D10–D36), (vi) mucinous cystadenocarcinoma of the pancreas (C259, C00–D48, C15–C26), (vii) mucinous cystadenoma of the pancreas (D136, C00–D48, D10–D36), (viii) neoplastic pancreatic cyst (D377, C00–D48, D37–D48), (ix) serous cystic tumor of the pancreas (D377, C00–D48, D37–D48), (x) mucinous cystic tumor of the pancreas (D377, C00–D48, D37–D48], and (xi) pancreatic cyst (K862, K00–K93, K80–K87), were automatically selected by the electronic medical record system (n = 2152). After excluding patients without IPMN (n = 499) and those without detailed medical information (n = 8), 1645 patients with IPMN were finally enrolled in the current clinical study (**Supplementary Figure S1**). All the patients were followed until February 29, 2024, or death, whichever came earlier. Patients with IPMN were classified into the following three different morphological phenotypes. First, the "branch-duct IPMNs" are defined as unilocular or multilocular pancreatic cystic lesions that communicate with the main pancreatic duct (MPD). Second, the "main-duct IPMNs" are defined as segmental or diffuse dilatation of the MPD of >5 mm without other causes of the MPD dilatation. Third, the "mixed-type IPMNs" are defined as lesions meeting the diagnostic criteria for both branch-duct and main-duct IPMNs[7].

Surveillance of IPMNs for the ascertainment of pancreatic carcinoma cases

All the patients underwent routine laboratory examinations, including general

biochemistry test, carcinoembryonic antigen (CEA), and carbohydrate antigen 19-9 (CA19-9) every 6–12 months based on the previous IPMN international consensus guideline[12]. The normal upper limit of the serum CA19-9 and CEA concentrations in the study was 37 U/mL and 5.0 ng/mL, respectively. Imaging tests were also performed, including MRI and/or contrast-enhanced CT. In case of any signs suspicious for pancreatic carcinoma development on these imaging modalities, endoscopic ultrasound-guided fine-needle aspiration (EUS-FNA) and/or endoscopic retrograde cholangiopancreatography (ERCP) were performed to confirm cytological or histological diagnosis of pancreatic carcinoma[13]. EUS-FNA was not performed in principle for the cytological analysis of the cyst fluid based on the local consensus in Japan[12]. Not only invasive carcinoma but also IPMN with high-grade dysplasia were regarded as malignant tumors and analyzed as IPMN-related carcinoma. In patients with IPMN-related carcinoma, those with IPMN-DC and concomitant PDAC were differentiated based on the radiological and/or pathological results.

Evaluation of the morphological features of IPMNs in the prediagnostic stage of pancreatic carcinoma

Based on the current international consensus guideline[11], we characterized the morphologic features of IPMNs before achieving a pancreatic carcinoma diagnosis focusing on WFs and HRS. To be specific, the former includes (i) an IPMN size ≥30 mm, (ii) an enhancing mural nodule <5 mm, (iii) a thickened enhanced cyst wall, (iv) an MPD diameter of 5–9.9 mm, (v) an abrupt caliber change of the MPD with distal pancreatic atrophy, (vi) lymphadenopathy, (vii) a cyst growth rate ≥2.5 mm/year, (viii) acute pancreatitis, (ix) increased serum CA19-9 level (normal upper limit, 37 U/mL), and (x) new onset or acute exacerbation of diabetes within the past 1 year, and the latter includes (i) obstructive jaundice in a patient with IPMN at pancreatic head, (ii) an enhancing mural nodule ≥5 mm or solid component, (iii) MPD diameter ≥10 mm, and (iv) suspicious or positive results of cytology (if, performed option). A further examination via endoscopic ultrasound is recommended for IPMNs harboring WFs and surgical

resection for IPMNs harboring HRS.

Ethical issues

This study was conducted following the Declaration of Helsinki and the principles of the Japanese ethics guideline for life science and medical research involving human subjects (https://www.mext.go.jp/lifescience/bioethics/files/pdf/n2373_01.pdf [Only Japanese text available]. Accessed on Mar. 28, 2025). Furthermore, the Ethical Committee of Nara Prefecture General Medical Center approved this study (approval number: 983). Informed consent was obtained by using an opt-out method.
