# Supplementary material for: Clinical Features of Intraductal Papillary Mucinous Neoplasm-Related Pancreatic Carcinomas in Long-Term Surveillance
Source: J Clin Med. 2025 Jun 27;14(13):4585. doi: 10.3390/jcm14134585 (PMC12249908; doi:10.3390/jcm14134585)
Supplement: Supplementary file 1 [file jcm-14-04585-s001.zip › Supplementary Table S1.pdf]

Supplementary Table S1. Clinical tumor stage of pancreatic neoplasms in patients with IPMN

Clinical tumor stage (UICC TNM 8th edition)	Whole cases (n = 164)	IPMN-DC (n = 123)	Concomitant PDAC (n = 41)
**Stage I**	48.8% (80/164)	52.0% (64/123)	39.0% (16/41)
**Stage II**	40.9% (67/164)	43.9% (54/123)	31.7% (13/41)
**Stage III**	3.0% (5/164)	0.8% (1/123)	9.8% (4/41)
**Stage IV**	7.3% (12/164)	3.3% (4/123)	19.5% (8/41)

IPMN-DC, intraductal papillary mucinous neoplasms derived carcinoma; PDAC, pancreatic ductal adenocarcinoma; UICC, union for international cancer control.
