# Supplementary figures and images for: Clinical Features of Intraductal Papillary Mucinous Neoplasm-Related Pancreatic Carcinomas in Long-Term Surveillance
Source: J Clin Med. 2025 Jun 27;14(13):4585. doi: 10.3390/jcm14134585 (PMC12249908; doi:10.3390/jcm14134585)

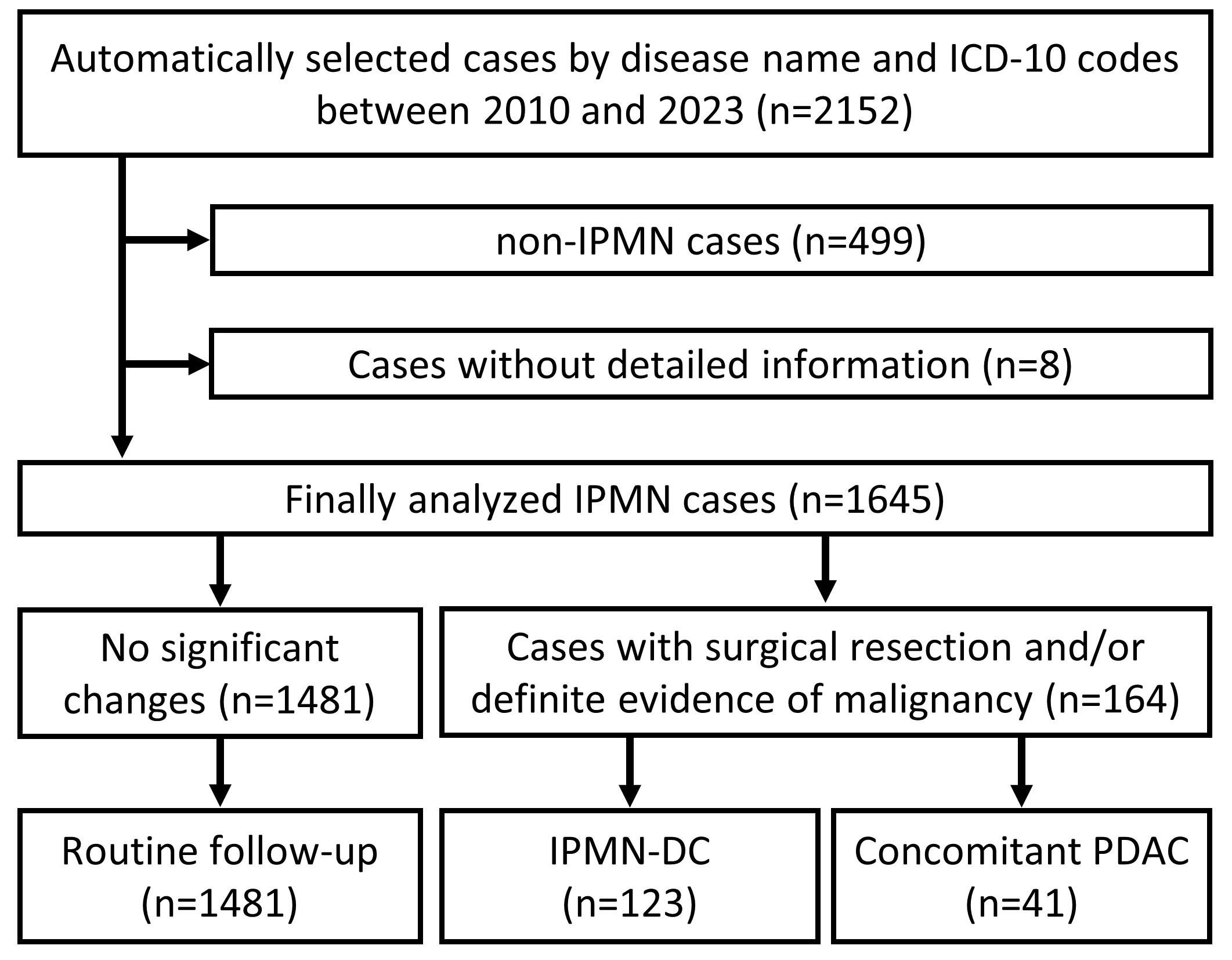

Supplement: Supplementary file 1 [file jcm-14-04585-s001.zip › Figure S1.tif]

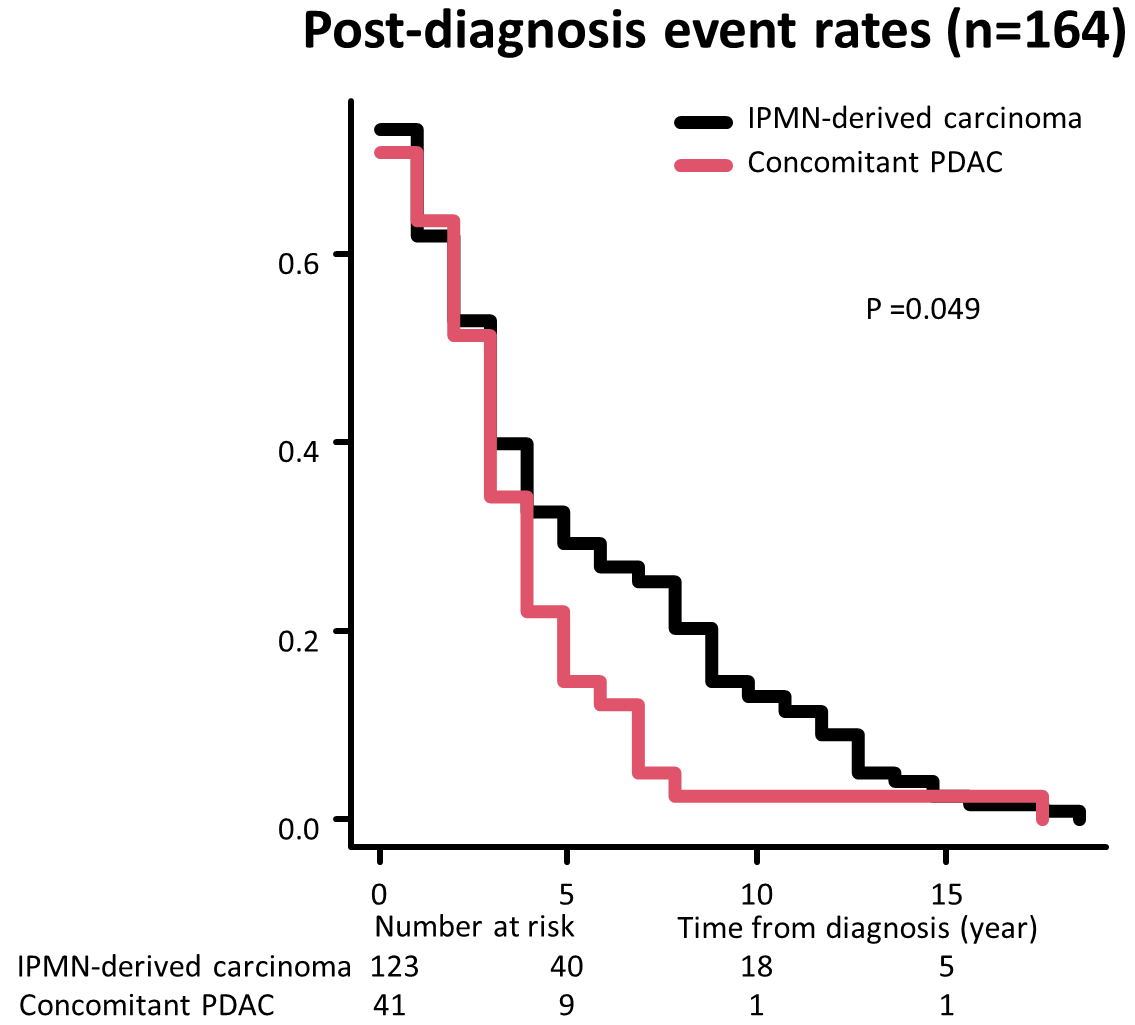

Supplement: Supplementary file 1 [file jcm-14-04585-s001.zip › Figure S2.tif]

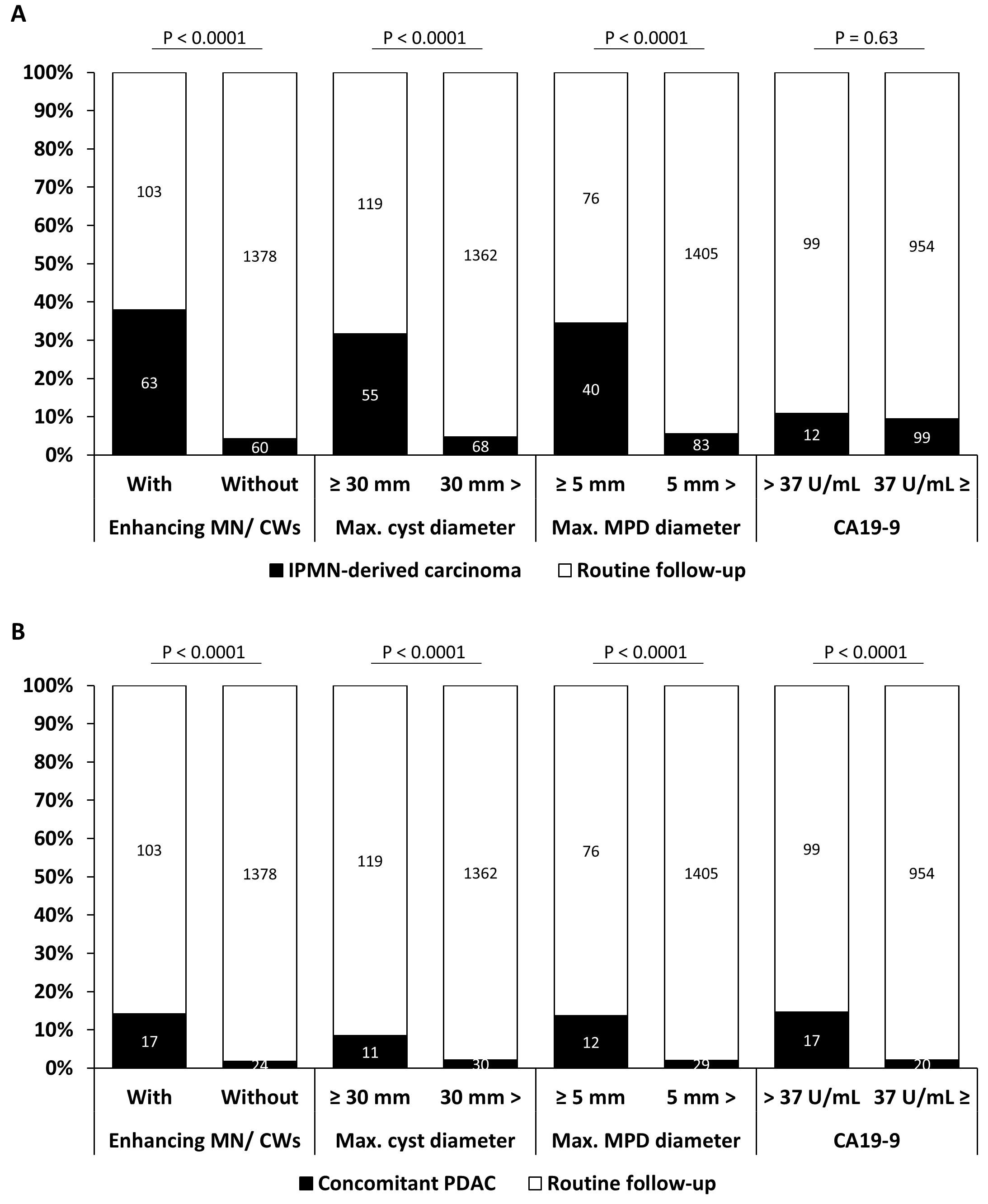

Supplement: Supplementary file 1 [file jcm-14-04585-s001.zip › Figure S3.tif]
